# Analysis of bacterial diversity in two oil blocks from two low-permeability reservoirs with high salinities

**DOI:** 10.1038/srep19600

**Published:** 2016-01-20

**Authors:** Meng Xiao, Shan-Shan Sun, Zhong-Zhi Zhang, Jun-Ming Wang, Long-Wei Qiu, Hua-Yang Sun, Zhao-Zheng Song, Bei-Yu Zhang, De-Li Gao, Guang-Qing Zhang, Wei-Min Wu

**Affiliations:** 1 State Key Laboratory of Heavy Oil Processing, China University of Petroleum, Beijing, 102249, P. R. China; 2 College of Chemical Engineering, Qingdao University of Science and Technology, Qingdao, 266042, P. R. China; 3 Dalian design branch, China Petroleum Engineering & Construction Corporation, Dalian 116011, P. R. China; 4 School of Geosciences, China University of Petroleum, East China, Qingdao, 266555, P. R. China; 5 College of Petroleum engineering, China University of Petroleum, Beijing, 102249, P. R. China; 6 School of Mechanical, Materials & Mechatronic Engineering, University of Wollongong, Wollongong, NSW2522, Australia; 7 Department of Civil and Environmental Engineering, William & Cloy Codiga Resource Recovery Research Center, Center for Sustainable Development & Global Competitiveness, Stanford University, Stanford, California, 94305-4020, USA

## Abstract

The community diversities of two oil reservoirs with low permeability of 1.81 × 10^−3^ and 2.29 × 10^−3^ μm^2^ in Changqing, China, were investigated using a high throughput sequencing technique to analyze the influence of biostimulation with a nutrient activator on the bacterial communities. These two blocks differed significantly in salinity (average 17,500 vs 40,900 mg/L). A core simulation test was used to evaluate the effectiveness of indigenous microbial-enhanced oil recovery (MEOR). The results indicated that in the two high salinity oil reservoirs, one reservoir having relatively lower salinity level and a narrow salinity range had higher bacterial and phylogenetic diversity. The addition of the nutrient activator increased the diversity of the bacterial community structure and the diversity differences between the two blocks. The results of the core simulation test showed that the bacterial community in the reservoir with a salinity level of 17,500 mg/L did not show significant higher MEOR efficiency compared with the reservoir with 40,900 mg/L i.e. MEOR efficiency of 8.12% vs 6.56% (test p = 0.291 > 0.05). Therefore, salinity levels affected the bacterial diversities in the two low permeability oil blocks remarkably. But the influence of salinity for the MEOR recovery was slightly.

Petroleum hydrocarbon resources with a large scale, high permeability and high abundance that are easy to explore and exploit have been remarkably reduced since the 20^th^ century due to tremendous oil production. In recent years, hydrocarbon resources with low abundance and low economic benefits are becoming the main exploration and exploitation targets in China, which has numerous low permeability petroleum resources. Low permeability reservoirs exhibit a number of characteristics including low porosity, small pore throat size, low fluid permeability, and low productivity, which make it difficult to produce economic volume of petroleum hydrocarbons. A number of exploitation techniques based on water flooding, including fracturing, well pattern optimization, advanced water injection, and horizontal drilling have been developed in order to produce economic volumes of petroleum hydrocarbons from low permeability reservoirs. However, the high water content after water flooding has become a severe problem. Thus, improving the oil recovery ratio of low-permeability reservoirs under high water content conditions is an urgent issue.

Microbial enhanced oil recovery (MEOR) has been proposed as a tertiary oil recovery technique due to its simplicity, wide applicability, and economic and environmental benefits. One widely tested MEOR is indigenous microbial-enhanced oil recovery (IMEOR), which stimulates the metabolism of microorganisms indigenous to oil reservoirs to enhance oil recovery. For this technique, air was successfully injected into the water with a nutrient activator to maintain the growth and metabolism of the aerobic microbes^1^. This method decreases the operational cost significantly and does not require microbial culture incubation above ground^2^. The indigenous microbial community is formed during the long flooding process and involves various microbes with functions that enhance oil recovery. Dynamic changes in the abundance in the community have been observed during all exploration periods^3^. A number of studies have focused on the diversity of microbial communities, environmental factor (temperature, salinity, permeability, etc.), and influence of nutrient injection. The major microbial species in oil reservoir always include mesophilic microorganisms, sulfate-reducing bacteria, denitrifying bacteria, methanogens, and so on^4^,^5^,^6^,^7^. The structure of the microbial community is influenced by oil reservoir geological conditions or external factors (nutrient injection, water flooding). For example, Lin *et al*. studied microbial communities in oil reservoirs that were developed by water flooding^8^. The structure of microbial community changed remarkably with the increase of formation temperature^8^,^9^. The higher the temperature was, the less species in formation water would be. Salinity is another influencing factor for microbial community structure. Wang *et al*. found that *in situ* nutrient injection had impact on MEOR and microbial diversity^10^. The microbial diversity in low salinity level oil reservoir was much more abundant than that in high salinity level oil reservoir^8^. After nutrient injection, some advantaged microbes, such as *pseudomonas, Ochrobactrum, Alcaligenes* and some methane producing archaea, had enormously increase^10^,^11^,^12^. The composition of nutrient determined what kind of species could be stimulated. Most microbial diversity analysis above was conducted in relatively high permeability reservoirs (89 × 10^−3^–6990 × 10^−3^ μm^2^). However, the bacterial community structure of a lower permeability reservoir with high salinity during the MEOR stimulation process have not been reported.

In this study, the distribution of bacteria in two low-permeability oil blocks (Wangyao block and Liu block, Changqing, China) was characterized using high throughput sequencing techniques to determine the influence of salinity. The succession of bacterial diversity of the two formation water samples after stimulation was also analyzed. We used a core simulation experiment to evaluate the performance of bacterial oil recovery and the influence of different permeability and salinity after stimulation.

## Results

### Geological characterization of the test reservoir blocks

The Changqing Oilfield is located in the Ordos Basin, which covers 370,000 km^2^ in Shanxi Province, northwest China, and is the second largest oil and gas field. In this oilfield, Block Wangyao and Block Liu have high connectivity and suitable geological conditions for the MEOR. The reservoir temperature is approximately 45 °C for Block Wangyao and 54 °C for Block Liu. These two blocks belong to an ultra-low permeability sandstone reservoir and have the same formation water type (CaCl_2_) and similar high salinity content (ranging from 10,800 mg/L to 80,560 mg/L). For Block Wangyao, the average buried depth is 1100–1300 m with a reservoir thickness of approximately 18.3 m, average porosity of approximately 13.7%, and permeability of approximately 2.29 × 10^−3^ μm^2^. Block Liu has an average buried depth of 1850 m with an average porosity of 12.69% and permeability of 1.81 × 10^−3^ μm^2^. Formation water samples were taken from one injection well and three production wells for each block. The characteristics of each water sample are described as in Table 1.

### Characterization of bacterial community diversity in a low permeability reservoir

#### (1) DNA isolation, sequencing and contig assembly

Using the bacterial V6 primer and paired-end model of the Illumina HiSeq2000 sequencer, we obtained a total of 4,308,143 merged reads after trimming primers and barcodes. The average number of merged reads for each sample was 538,518, with a length of 200 bp and GC content of 53%. Reads that had similarities above 97% were clustered into the same OTUs (Operational taxonomic unit) to calculate the rarefaction and analyze the taxonomic richness and diversity.

#### (2) Species diversity among different samples

**Figure 1:**
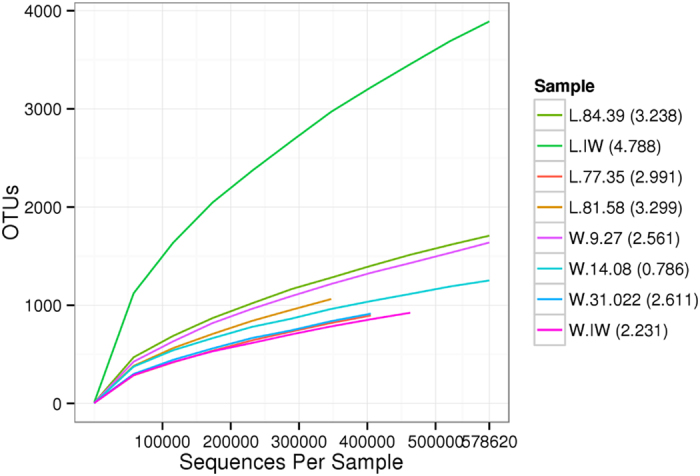
Rarefaction analysis for each sample. The rarefaction curves in the figure represent the OTUs vs sequences in each sample. Numbers in parentheses in the legends are the Shannon indices of each sample.

The rarefaction curves (Fig. 1) indicated that the sequencing results had good coverage of the species in the 8 water samples. The Shannon index of bacterial diversity was calculated using the data of the samples withdrawn to assess the evenness of the OTU distribution. The eight formation water samples had different Shannon indices, indicating that the eight formation samples were different (Fig. 1). The Shannon indices of the four samples from Block Liu were higher than those of Block Wangyao. The sample L.IW had the highest Shannon index, indicating that the highest bacterial diversity was present in the injection water of Block Liu; in contrast, the index of W.IW was only 2.231. Sample W.14.08 had the lowest Shannon’s index (0.786). These results suggested that the bacterial diversity of Block Liu was richer than Block Wangyao.

#### (3) Influence of salinity on microbial genetic homology

**Figure 2:**
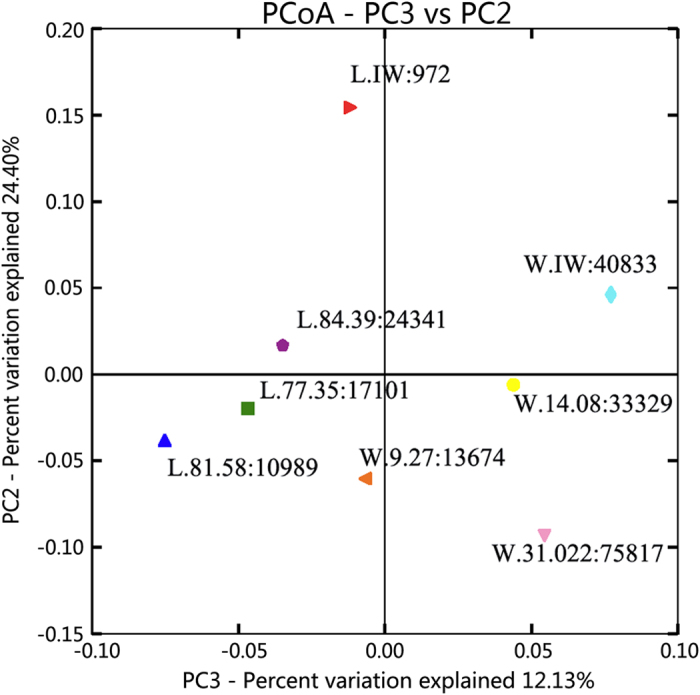
PCoA for the relationship between salinity and bacterial diversity in the two blocks. The salinity values of each sample were labeled after each sample ID.

The relationship between salinity and bacterial diversity was shown in Fig. 2. The salinity differed between the wells of the two blocks, ranging from 10,989 mg/L to 75,817 mg/L. Sample L.IW, which consisted of injection water from Block Liu, contained the lowest salinity (972 mg/L) (Table 1 and Fig. 2); moreover, its diversity had the largest distance from the other 7 samples, which suggests that the difference in salinity could have a remarkable effect on the bacterial diversity between the injection water and formation water. Sample L.IW with the lowest salinity had the highest bacterial diversity based on its Shannon Index (Fig. 1). Figure 2 shows that the samples L.84.39, L.81.58 and L.77.35 clustered much more compactly, which implied phylogenetic diversity; their salinities ranged from 10,989 mg/L to 24,341 mg/L. However, samples W.14.08, W.9.27 and W.31.022 had lower phylogenetic diversity because the distances among these samples were longer than the distances among samples L.84.39, L.81.58 and L.77.35. The salinity of these samples varied within a large range (13,674 mg/L to 75,817 mg/L).

#### (4) Taxonomic analysis and distributions of functional microbes in the oil reservoir

**Figure 3:**
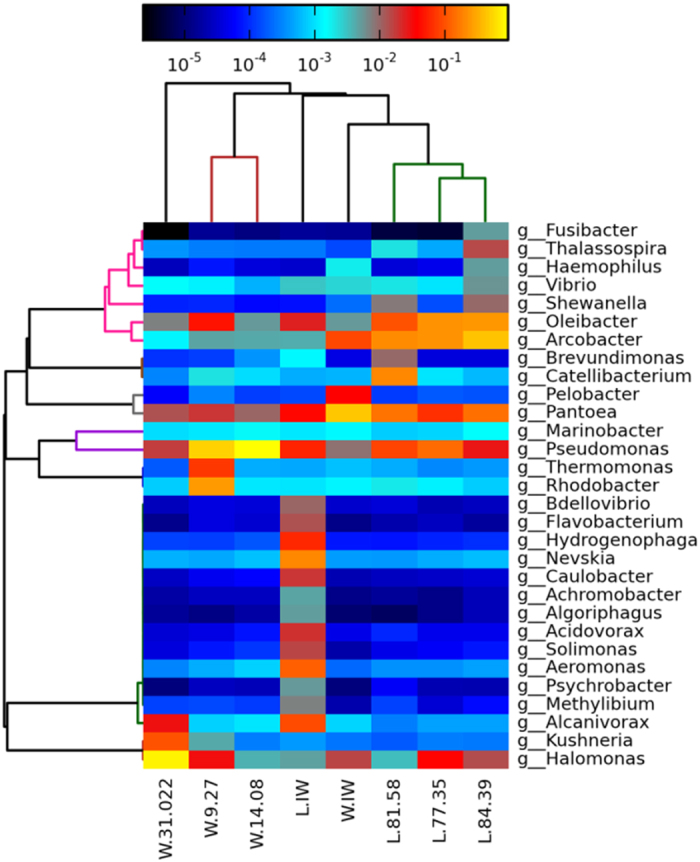
Phylogenetic analysis of eight formation water samples at the genus level. The black to yellow colors indicate low to high representation of OTUs.

All sequences were classified from phylum to genus. The eight samples had common species at each taxonomic level. The main species at the phylum level was *Proteobacteria* (Supplementary Fig. S1). As shown in Fig. 3, *Halomonas, Pantoea, Pseudomonas*, and *Oleibacter* had high abundances at the genus level in the eight water samples. Because *Halomonas* had a high tolerance to high salinity, its relative abundance was highest in sample W.31.022, which had the highest salinity (75,817 mg/L). The abundance of *Halomonas* was also considerable in the low salinity samples W.9.27, L.77.35 and L.84.39. This finding is consistent with the previous report that *Halomonas* was found in the groundwater of a hydrocarbon-contaminated site with a high level of salinity^13^. *Halomonas eurihalina* can also produce metabolites (i.e., extracellular polysaccharides) to emulsify crude oil^14^. *Pantoea* is considered an anaerobic hydrocarbon-degrading bacterium that has the ability to produce rhamnolipid and metabolite paraffin, diesel and other hydrocarbons^15^. The production of the biosurfactant could improve the hydrophobic properties of the cell surface and enhance the metabolism of hydrocarbons. Bhatia reported that the sulfur content of crude oil was reduced by 26.38%−71.42% by the desulfurization of *P. agglomerans* D23W3^16^. *Oleibacter* is a hydrocarbon-degrading bacterial species that exhibits high n-alkane-degrading activity^17^,^18^. *Pseudomonas* widely exists in oil reservoirs, including the typical species *Pseudomonas fluorescens, Pseudomonas aeruginosa*, and *Pseudomonas stutzeri*. These organisms have several functions related to crude oil exploitation and the petroleum industry, such as emulsion, hydrocarbon degradation, rhamnolipid metabolism and representation in oil recovery and environmental contamination fields^15^,^19^,^20^. Rhamnolipids could improve the hydrophobicity of microbial cells, resulting in a lower surface tension and an increased adhesion ratio of cells onto hydrocarbons, which could enhance the metabolism of crude oil.

In addition to the highly abundant species in common between the samples discussed above, two blocks had their own distinct dominant species. *Arcobacter, Shewanella* and *Thalassospira* were plentiful in Block Liu, while *Thermomonas, Rhodobacter, Alcanivorax* and *Kushneria* were dominant in Block Wangyao. *Shewanella, Thalassospira* and *Alcanivorax* are common in aqueous environments and possess hydrocarbon-degrading capabilities^21^,^22^. *Alcanivorax* would exhibit a large scale propagation when nitrogen and phosphorous sources were sufficient, and *Alcanivorax* was reported to play an important role in the bioremediation of oil-contaminated marine environments^23^. In the injection water of Block Liu, the major species belonged to marine microbes, which have a high salt tolerance and are capable of producing biosurfactants. Therefore, the injection water could provide functional microbes for oil recovery, including *Oleibacter, Pantoea* and *Pseudomonas*. The oil reservoir water also had its own indigenous functional microbes (*Kushneria, Halomonas, Thermomonas, Pelobacter*, and *Arobacter*) that are capable of metabolizing hydrocarbons, indicating that the two blocks had great potential for indigenous microbial-enhanced oil recovery.

#### (5) Community diversity changes after stimulation

**Figure 4:**
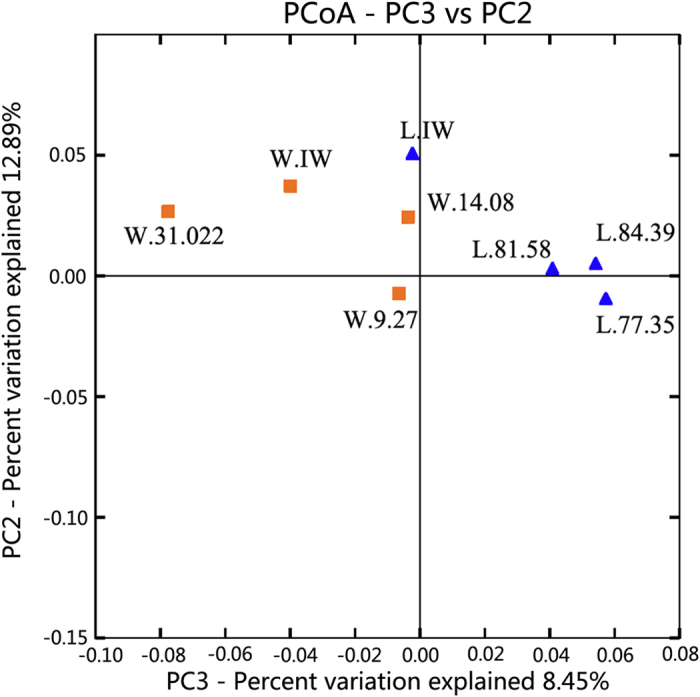
PCoA for indigenous microbial diversity of the two blocks. ■ Samples in Block Wangyao; ▲ Samples in Block Liu.

**Figure 5:**
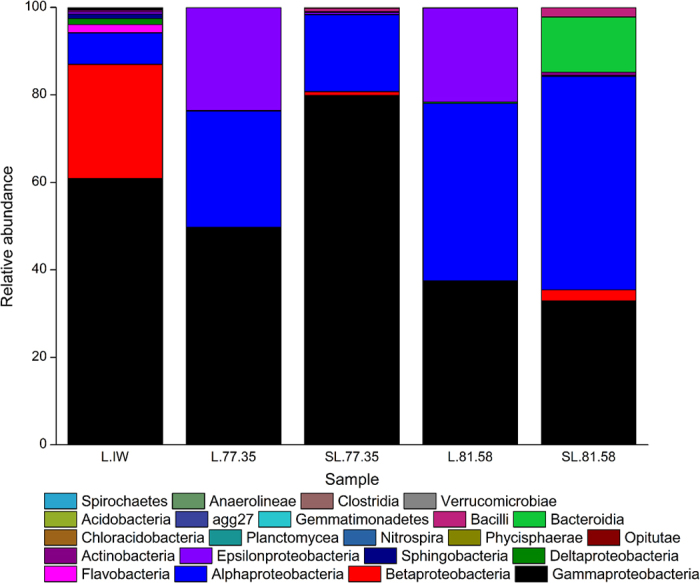
Comparison of bacterial diversity before and after stimulation by the addition of the activator at the class level. L.IW was injection water; L.77.35 and L.81.58 were initial water samples from oil production wells; and SL.77.35, SL.81.58 were the samples after stimulation.

**Figure 6:**
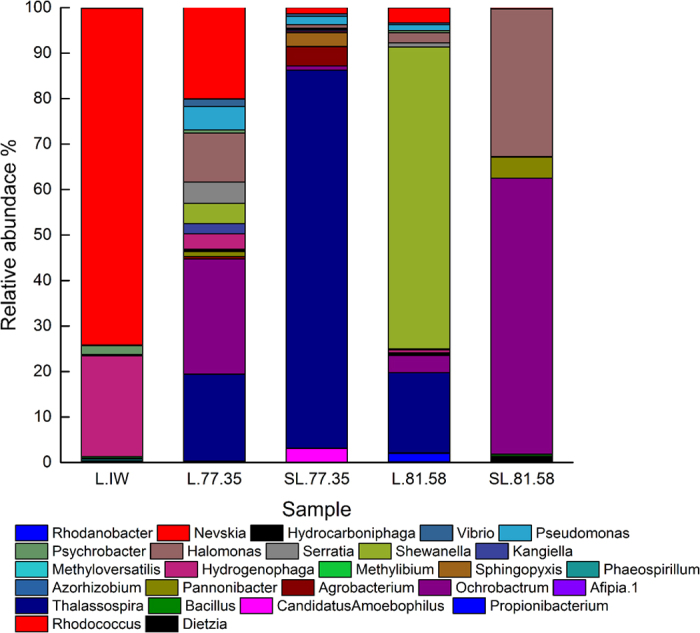
Comparison of the dominant bacteria before and after the stimulation by the addition of activator at the genus level. L.IW, L.77.5 and L.81.58 were the initial water samples of the oil well, and SL.77.35 and SL.81.58 were stimulated production water samples.

In this study, the microbial activities of samples L.77.35 and L.81.58 were stimulated by adding a nutrient activator; both samples were located in close proximity in the same block (Fig. 4). The variation in the bacterial communities was shown by the Beta diversity matrix analysis (Supplementary Information, Fig. S2). The distance between L.81.58 and SL.81.58 was larger than the distance between L.77.35 and SL.77.35. Compared with the Shannon indices of L.77.35 (2.991) and L.81.58 (3.299), the Shannon indices of SL.77.35 and SL.81.58 increased to 4.094 and 4.273, respectively. This result indicates a significant change in the microbial community of L.81.58. The variation in the dominant species abundance at the class level was shown in Fig. 5 and at the genus level in Fig. 6. The microbial species was richer in the injection water compared to the two production water samples. The dominant species in sample L.IW were *Gammaproteobacteria, Betaproteobacteria* and *Alphaproteobacteria* (Fig. 5). The representative species were *Nevskia, Hydrogenophaga* and *Phaeospirillum*. After injection into the oil reservoir, the production waters of L.77.35 and L.81.58 were dominated by *Gammaproteobacteria, Alphaproteobacteria* and *Epsilonproteobacteria* with the representative bacteria *Nevskia, Ochrobactrum, Thalassospira, Halomonas, Pseudomonas*, and *Shewanella*.

During the stimulation treatment, *Betaproteobacteria* appeared again in the two stimulated samples. In sample SL.81.58, *Gammaproteobacteria* was slightly decreased, while *Alphaproteobacteria* become the species with the largest abundance. *Bacillus, Bacteroidia* and *Actinobacteria* were also present. Some species showed a high or increased abundance. For example, *Ochrobactrum* and *Halomonas* had improved abundances of 60.61% and 32.47%, respectively. *Dietzia* and *Pannonibacter* were present with abundances of 1.38% and 4.63%, respectively. *Bacillus* also appeared with an abundance of 0.37%. However, the abundances of *Shewanella, Thalassospira, Nevskia, Propionibacterium* and *Pseudomonas* were decreased after the stimulation treatment. The genus *Shewanella* has been found in many aqueous environments, including the deep sea^24^. *Thalassospira* and *Pseudomonas* are commonly found in petroleum environments^25^. In sample SL.77.35, the abundance of *Gammaproteobacteria* was highly increased. Bacteria with an increased abundance were *Thalassospira, Agrobacterium, Candidatus, Amoebophilus* and *Sphingopyxis*. The abundances of *Ochrobactrum, Halomonas, Pannonibacter* and *Dietzia* were reduced to different degrees after stimulation.

### Core simulation experiment

The results of the core simulation experiments are summarized in Table 2. The air permeability of Block Liu (3.14 × 10^3^–6.06 × 10^3^ μm^2^) was similar with Block Wangyao (3.04 × 10^3^–6.13 × 10^3^ μm^2^). The oil final efficiency and average improvement efficiency of Block Liu were higher than those of Block Wangyao. However, the MEOR value was increased slightly by 8.12% in Block Liu, which was slightly higher than that in Block Wangyao (6.56%). According to t-test result (p = 0.291 > 0.05), MEOR efficiencies of these two blocks did not show significant difference. Therefore, in a reservoir with high salinity (17,500–40,900 mg/L) and low permeability (3 × 10^−3^–6 × 10^−3^μm^2^), both salinity and permeability may not be a significant factor for MEOR when the indigenous bacterial had been stimulated. This also indicated that the diversity differences between the two blocks may not be associated with the MEOR efficiency.

## Discussion

The bacterial diversity distribution was analyzed in two oil blocks with different formation conditions. The results indicated that the two blocks had distinct bacterial community structures. Based on the Shannon index analysis, Block Liu had a higher species diversity that was probably caused by the invasion of exogenous species in the injection water, which came from surface water and had low salinity (972 mg/L) and high microbial diversity. In contrast, sample W.IW had high salinity (40,883 mg/L) with relatively low microbial diversity because the original source was the treated oilfield water for recycling use.

The difference among the bacterial communities of the formation water was determined by the Beta diversity analysis, which represented the external comparison among samples by Principal Coordinate Analysis (PCoA). As shown in Fig. 4, samples L.84.39, L.73.35 and L.81.58 were clustered into one group and samples W.IW, W.31.022, W.9.27 and W.14.08 were clustered into a second group. This indicated that the bacterial communities in the two blocks significantly differed from each other and that the communities in the wells within the same block showed high similarity in bacterial species compositions. The taxonomic analysis by phylogenetic analysis (Fig. 3) indicated that samples W.9.27 and W.14.08 in Block Wangyao and samples L.77.35 and L.84.39 clustered together, representing the highest homology among the eight samples. The species differences between sample W.IW and the other samples in Block Wangyao were not remarkable because the oilfield injection water in Wangyao Block was the treated formation water used for recycling and the environment of the injection water for bacteria was not changed remarkably. However, the differences between sample L.IW and the other samples of Block Liu were significant (Fig. 4). This result indicated that although foreign species in surface water could be introduced into the oil formation through injection wells and influence the diversity of the bacterial community, the difference between the injection water and formation water was still significant. The redox state of the surface water would change from oxic to anoxic during the flooding process and contribute to the obvious shift in the microbial community. Lenchi *et al*.^26^ reported a significant difference in the microbial compositions in the injection and production waters because most of the bacteria in the injection water were not retrieved in the production water. Thus, the introduction of surface water could improve the microbial diversity of the formation water but could not change the major bacterial community structure or the bacterial composition in the reservoir environment. In other words, when foreign species invaded the oil reservoir, they could adapt to the reservoir environment. However, the extreme environment (i.e., high salinity, relatively high temperature and high pressure) depressed the growth of these foreign species and selected the species that survived well, resulting in a relatively stable indigenous bacterial ecosystem.

The microbial community structure was affected by temperature, mineralization, ionic type and hydrocarbon content. Many researchers have reported the influence of temperature. Zhang *et al*.^9^ reported that the effects of microorganisms in the injected water on microbial community diversity in the production water decreased with the increase in temperature. An investigation performed by Wang *et al*.^27^ indicated that low temperature reservoirs grouped together in the PCoA analysis, but high temperature petroleum reservoirs did not group together due to large differences in mineralization and the Cl^−^ concentration. Salinity is one of the most important factors that influences microbial community diversity. In this study, the formation temperatures of the two Blocks were slightly similar (45 °C for Block Wangyao and 54 °C for Block Liu). Therefore, the influence of temperature was little. Table 1 showed that the salinity range of the production water in Block Liu was narrower than that in Block Wangyao. Samples L.77.35, L.81.58 and L.84.39 clustered much more closely compared with samples W.9.27, W.14.08 and W.31.022 (Fig. 4), thus indicating higher phylogenetic diversity. Therefore, the salinity distribution range could affect the phylogenetic diversity of the bacterial community, even if they were in the same area. The larger the range, the more genetic differences existed among the production water. Previous studies also illustrated that the microbial diversity was low in the hypersaline environment and declined as the salinity increased^28^,^29^. Wang *et al*.^28^ studied the relationship between salinity (ranging from 0.2 mg/L to 280 mg/L) and microbial community diversity and indicated that the bacterial community of Tibetan lakes with higher salinity had higher diversification than the communities of freshwater lakes, whereas the phylogenetic diversity in the hypersaline lake was lower compared to the freshwater lake. Moreover, salinity had a negative influence on subsurface archaeal diversity and methanogenesis in the Antrim Shale sedimentary region^30^. In this study, salinity influenced and selected the microbial communities of the two blocks. The Shannon indices of the low salinity samples L.77.35, L.81.58 and L.84.39 in Block Liu were higher than those of samples W.9.27, W.14.08 and W.31.022 with high salinity in Block Wangyao (Fig. 1). This result confirmed that the microbial diversification in Block Liu within a narrow salinity range environment was higher than the diversity in Block Wangyao with a wide salinity range. The distances among L.77.35, L.81.58 and L.84.39 were closer than the distances among W.9.27, W.14.08 and W.31.022, implying the presence of higher phylogenetic diversity in Block Liu compared to Block Wangyao. Therefore, salinity could affect the genetic relationship of indigenous microbes in the high salinity and low permeability oil reservoir, and a narrow range in the oil reservoir could be more favorable to bacterial and phylogenetic diversity in these blocks.

Changes in microbial community diversity appeared to have less impact on the MEOR performance based on the results of our simulation experiments (Table 2). The two initial samples had similar OTU numbers and Shannon indices (Fig. 1), which represented similar bacterial diversity. The two production wells were in the same block and had a similar formation temperature. After stimulation, the two stimulated samples exhibited remarkable differences compared with their original samples (Fig. S2). Species capable of producing surfactants, such as *Pseudomonas, Ochrobactrum, Acinetobacter* and *Halomonas*, were enriched in sample SL.81.58, which was similar to the findings reported by Gao *et al*.^11^. As well, the abundance of bacteria in the two samples differed remarkably. The addition of the nutrient activator probably changed the microbial community structure and increased the microbial diversity, even though the samples were located in the same block. To improve oil recovery, the composition of the nutrient activator should be adjusted according to the changes in the functional bacterial community, especially for the hydrocarbon-degrading, biosurfactant-producing bacteria, for each block or oil well.

Using core simulation test, the difference in MEOR efficiencies between the two blocks was limited (8.12% for Block Liu versus 6.56% for Block Wangyao). The improvement values of L6 (31.27%) and W8 (23%) were higher than L10 (29.99%) and W10 (18%) (Table 2). This finding suggested that the MEOR improvement with lower air permeability might be higher than the value with higher water phase permeability. Microbes can transport freely in the low-permeability pores, Bryant reported that some microorganisms might exhibit better recovery efficiency in lower permeability (0.134 μm^2^–1.92 μm^2^) cores^31^. Therefore, the core permeability may not have a remarkable influence on the MEOR process in the two blocks. The nutrients injection enhanced indigenous microbes’ reproduction and the metabolites production. As a result, the transport of crude oil likely benefited from microbes and the displacement of metabolites in the porous environment. Thus, in high salinity and low permeability oil reservoir, the influence of permeability on oil recovery was not remarkable when the indigenous microbes had been stimulated, although the salinity level and range might be the predominant influence factor for the initial bacterial diversities.

In conclusion, the bacterial diversities in different blocks demonstrated remarkable differences in the low-permeability reservoirs tested. The salinity level and range in high salinity reservoir affected the bacterial and phylogenetic diversity. In high salinity and low permeability oil reservoir, a block with a relatively low salinity level and a narrow salinity range could have high bacterial and phylogenetic diversity. The bacterial community structure was changed during the stimulation process by the addition of nutrients. The core simulation test indicated that, permeability is not a predominant factor for MEOR. The bacterial community in a narrow salinity environment had insignificantly higher oil recovery efficiency than that the community in the wide salinity range environment after stimulation in low permeability oil reservoir.

## Methods

### Sample collection and preparation

The Changqing oil field has low permeability and high salinity. Two well groups from the Wangyao and Liu blocks were investigated as target test wells in this study. For each group, three formation water samples and one injection well water sample were collected and immediately preserved in 15-L plastic containers. All containers were autoclaved and rinsed with the water sample to be collected prior to sampling. To avoid oxygen exposure, the containers were filled with the water sample and then closed. Subsequently, all containers were transported in a cooler filled with ice blocks and stored at 4 °C prior to DNA extraction. To separate the crude oil from the water sample, the sample was kept at 70 °C for 15 h^32^. Then, the separated water was filtered through a 2.7-μm glass filter (Whatman Ltd., Maidstone, UK) to remove impurities with a 57-mm glass chimney filter unit. Subsequently, microbes were separated from the filtrate by passing through a 0.22-μm cellulose acetate filter (Whatman Ltd., Maidstone, UK), and the filter was immediately frozen in buffer at −80 °C prior to DNA isolation^33^.

### Stimulation of indigenous microbes

Two samples chosen from each well group were used to investigate the changes in microbial diversity before and after stimulation. A nutrient activator was added to the selected formation water samples to stimulate the indigenous microbes. The organic nutrient activator contained (per L of formation water): 6 g molasses, 9 g NaNO_3_, 1.67 g (NH_4_)_2_HPO_4_, 0.02 g FeSO_4_·7H_2_O, 0.1 g MgSO_4_, and 0.0005 g MnSO_4_·H_2_O. Samples of formation water were placed in 150-mL Erlenmeyer flasks. All samples were cultured at 45 °C under shake-flask conditions (150 rpm). Each experiment was conducted in triplicate.

### DNA isolation, sequencing, and initial data processing

DNA from the microbes collected on the cellulose acetate filter was isolated using a PowerSoil DNA Isolation Kit (MO BIO Laboratories Inc., Carlsbad, California, USA) according to the manufacturer’s instruction. The hypervariable V6 region of the bacterial 16S rRNA gene was amplified with the primers 967F (5′-CAACGCGARGAACCTTACC-3′) and 1061R (5′-ACAACACGAGCTGACGAC-3′). To multiplex the samples during sequencing, barcodes were added to the 5′ termini of the forward primers. The polymerase chain reaction (PCR) amplification system for each sample consisted of the GoTaq Colorless Master Mix (Promega, Madison, WI), 20 ng of sample DNA and 25 pmol of each primer with a final volume of 25 μL. The PCR procedure was similar to that reported by Gloor *et al*.^34^, with an initial denaturation at 94 °C for 2 min, 10 cycles of denaturation for 1 min at 94 °C, annealing from 61 to 51 °C with 1 °C decrements for 1 min per cycle, extension at 72 °C for 1 min, followed by 20 cycles of denaturation at 94 °C, annealing at 51 °C, and extension at 72 °C (all for 1 min), and a final elongation for 2 min. Each PCR reaction was run in triplicate. The quality of the PCR products was assessed using the NanoDrop 1000 spectrophotometer (Thermo Fisher Scientific Inc, Wilmington, USA). Subsequently, the PCR products were purified with the E.Z.N.A. Cycle-Pure Kit (Omega Bio-Tek, Inc., Norcross, USA) according to the manufacturer’s instruction and were then mixed uniformly. The mixed PCR products of the 16S V6 region were sequenced using Hiseq2000 at the Beijing Institute of Genomics, Chinese Academy of Sciences.

### Bioinformatics analysis

A multimillion-sequence 16S rRNA hypervariable V6 region library from complex microbial communities was generated using the 101-bp PE strategy on the Illumina HiSeq 2000 according to the manufacturer’s instruction. Then, the raw data were filtered by the removal of the joints and low quality sequences to generate clean data, followed by trimming of the primer sequence from the beginning and end of the clean data. The FLASH method described by Magoč and Salzberg^35^ was used to merge the forward and reverse reads when a correct overlap was found. The tagged sequences were selected approximately 120 bp after the overlap, and their quality was evaluated using the Fast QC software, including data quality, sequence length, and guanine-cytosine (GC) content. Then, the sequences were classified into different files according to the sample barcodes^36^. OTU screening, taxonomic richness and diversity analysis were also performed as described by Caporaso *et al*.^34^. All sequences were assigned taxonomic affiliations with an assignment cutoff of 0.03. The Ribosomal Database Project (RDP) classifier was used to assign taxonomic data to each representative sequence. The phylogenetic analysis was performed using PyNAST.

### Core simulation experiment

Core simulation experiments are an effective approach to evaluate the enhancement of the oil recovery technique. In this study, controlled trials were performed to evaluate the IMEOR effect on the change in the indigenous microbial community structure using 12 natural cores from two well groups. These natural cores had similar permeability and porosity. The water used was the formation water collected from the two blocks, which was then filtered to remove large particles. Crude oil was collected from the two well groups and then diluted to the corresponding viscosity using kerosene to reach 1.93 mPa.s for the Wangyao block and 2.02 mPa.s for the Liu block.

The cores were injected into the formation water in a vacuum container, and then the pressure was elevated to 10–15 MPa for 3 d at 45 °C. The water phase permeability, pore volume and porosity were calculated. Subsequently, the formation water was displaced from these cores using crude oil in a core holder until the oil saturation reached the original oil saturation level. The cores were immersed and aged in the crude oil for 7d at 45 °C.

Based on the hydrogeological conditions of the two well groups, a water flooding experiment was conducted at a corresponding water driven velocity. First, all cores were driven by formation water to achieve the water content in which approximately 0.5 per volume C (PV) of formation water with activator was injected into the cores. Then, the core holder was closed and the cores were maintained at the target respective temperature for 10d to stimulate the indigenous microbes. After stimulation, the formation water was driven untile 5 PV. During the experiment, the water content was calculated per 0.5 PV. The procedure of the controlled experiments was the same as described above, except the driven phase contained only the formation water.

## Additional Information

**How to cite this article**: Xiao, M. *et al*. Analysis of bacterial diversity in two oil blocks from two low-permeability reservoirs with high salinities. *Sci. Rep.* **6**, 19600; doi: 10.1038/srep19600 (2016).

## Supplementary Material

Supplementary Information

## Figures and Tables

**Table 1:** Geological information of the two Blocks.

Block	Well ID	Oil production (t/day)	Water content (%)	pH	Temperature(°C)	Salinity (mg/L)	Average salinity (mg/L)
Liu	L.IW	—	—	7.2	54 ± 3.62	972	—
	L.77.35	4.65	40.00	7.6		17101	17477 ± 6683.94
	L.84.39	1.59	78.41	7.8		24341	
	L.81.58	2.35	85.14	7.9		10989	
Wangyao	W.IW	—	—	7.6	45 ± 4.38	40833	—
	W.31.022	1.31	75.90	7.5		75817	40940 ± 31762.93
	W.9.27	0.95	64.60	7.5		13674	
	W.14.08	0.48	78.00	7.4		33329	

Note: L: Block Liu; W: Block Wangyao; L.IW: Injection water of Block Liu; W.IW: Injection water of Block Wangyao.

**Table 2:** Results of the core simulation experiment with core samples from Block Liu and Block Wangyao.

Core ID	Length (cm)	Pore volume ( mL)	Porosity (%)	Oil saturation (%)	Air permeability (×10^−3^ μm^2^)	Water content after water flooding (56%)		Oil recovery			
						Oil displacement efficiency (%)	Injected media (0.5PV)	Final efficiency (%)	Improvement value (%)	Avage improvement value (%)	MEOR value (%)
L3	6.94	4.36	12.69	53.9	4.70	19.15	water	40.43	21.28	23.00 ± 1.72	/
L9	6.55	5.07	15.60	51.8	4.18	20.91		45.63	24.72		
L11	6.58	4.76	11.25	54.6	3.14	21.38		44.39	23.01		
L6	6.8	4.38	13.10	53.5	5.50	19.57	Bacteria solution	50.84	31.27	31.12 ± 1.07	8.12 ± 2.79
L10	7.64	4.76	12.49	54.7	6.06	26.65		56.64	29.99		
L13	6.76	4.93	11.87	53.85	4.18	21.85		53.96	32.11		
W11	7.46	4.45	12.05	47.2	5.85	23.11	Water	37.82	14.71	14.19 ± 0.64	/
W12	7.59	4.92	13.10	41.7	5.86	26.09		39.57	13.48		
W14	6.73	4.68	12.56	43.75	3.04	23.64		38.02	14.38		
W8	7.3	4.81	13.31	41.6	6.00	27.50	Bacteria solution	50.50	23.00	20.75 ± 2.54	6.56 ± 3.18
W10	7.64	4.75	12.56	42.1	6.13	30.00		48.00	18.00		
W13	6.93	4.80	12.72	45.91	3.60	26.42		47.67	21.25		
