# Supplementary material for: Analysis of bacterial diversity in two oil blocks from two low-permeability reservoirs with high salinities
Source: Sci Rep. 2016 Jan 20;6:19600. doi: 10.1038/srep19600 (PMC4726302; doi:10.1038/srep19600)
Supplement: Supplementary Information [file srep19600-s1.pdf]

Analysis of bacterial diversity in two oil blocks from two low-permeability reservoirs with high salinities

Meng Xiao[1, 2], Shan-Shan Sun[1,*], Zhong-Zhi Zhang[1], Jun-Ming Wang[3], Long-Wei Qiu[4], Hua-Yang Sun[2], Zhao-Zheng Song[1], Bei-Yu Zhang[3], De-Li Gao[5], Guang-Qing Zhang[6], Wei-Min Wu[7]

[1] State Key Laboratory of Heavy Oil Processing, China University of Petroleum, Beijing, 102249, P. R. China

[2] College of Chemical Engineering, Qingdao University of Science and Technology, Qingdao, 266042, P. R. China

[3] Dalian design branch， China Petroleum Engineering & Construction Corporation, Dalian 116011, P. R. China

[4] School of Geosciences, China University of Petroleum, East China, Qingdao, 266555, P. R. China

[5] College of Petroleum engineering, China University of Petroleum, Beijing, 102249, P. R. China

[6] School of Mechanical, Materials & Mechatronic Engineering, University of Wollongong, Wollongong, NSW2522, Australia

[7] Department of Civil and Environmental Engineering, William & Cloy Codiga Resource Recovery Research Center, Center for Sustainable Development & Global Competitiveness, Stanford University, Stanford, California, 94305-4020, USA

[*] Corresponding author (Email: sssun_edu@163.com)

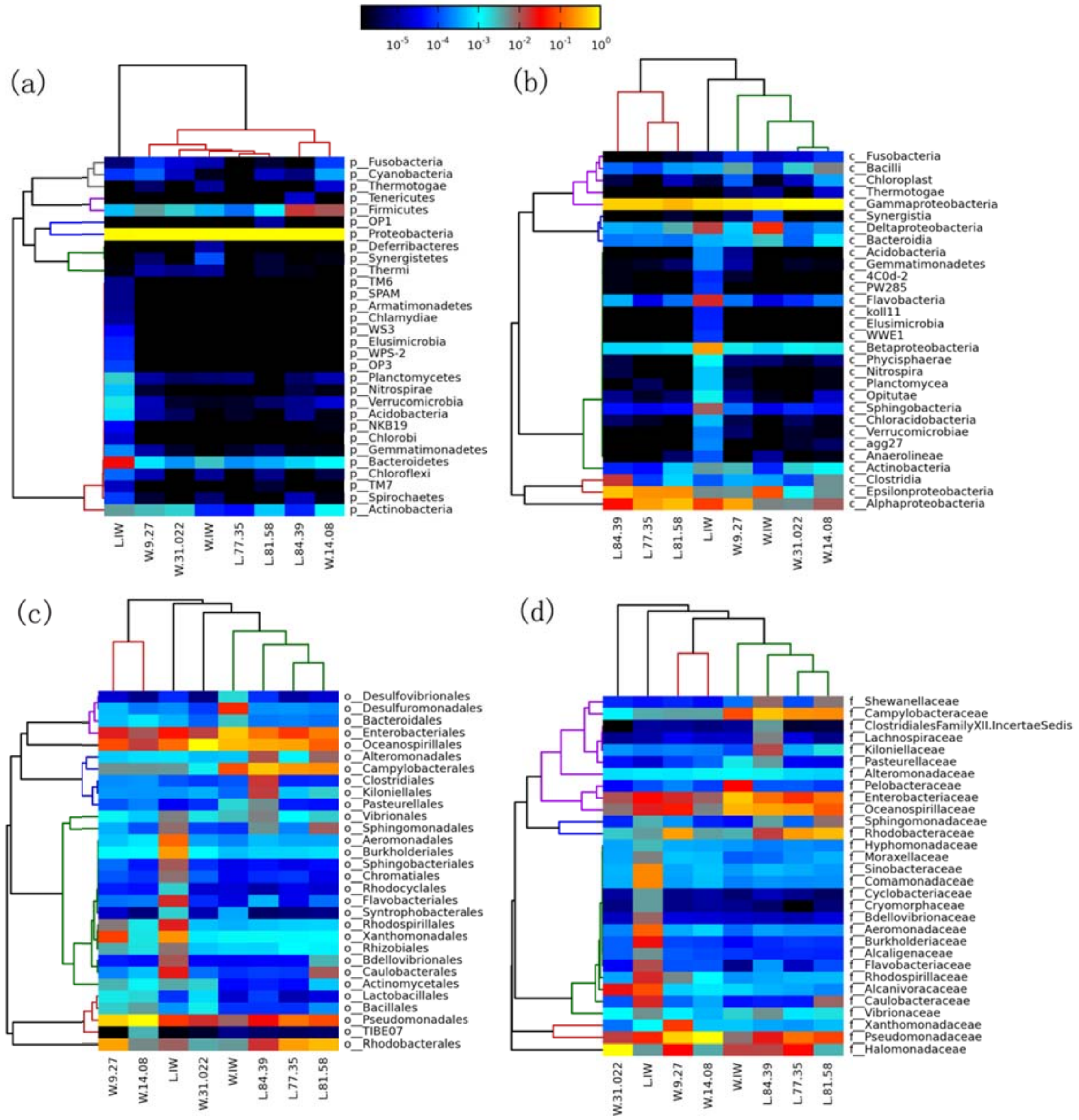

Fig. S1 Phylogenetic analysis of eight formation water samples at different taxonomic levels. Black to yellow color indicates low to high representation of OTUs. a. Cluster graph at phylum level; b. Cluster graph at class level; c. Cluster graph at order level; d. Cluster graph at family level.

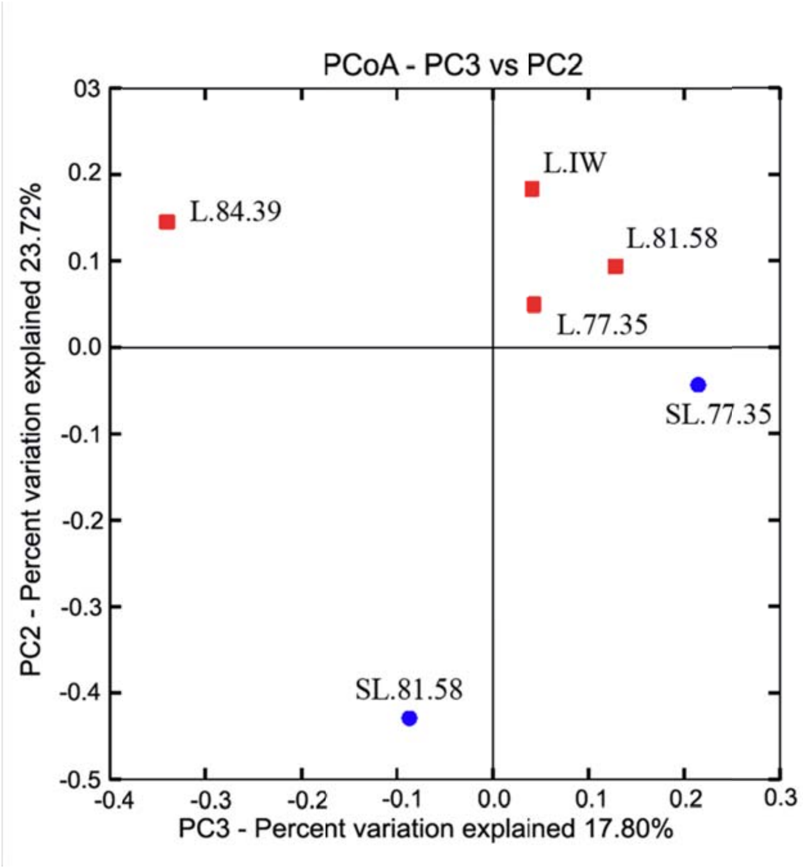

Fig. S2 Beta diversity analysis for the stimulation samples.

The red cycles are initial samples without biostimulation; the blue cycles are the stimulated samples.

Table S1 relative abundance of the major species before and after stimulation by additing activator nutrients in sample 77.35 (%)

	L.77.35	SL.77.35
Agrobacterium	0.51	25.30
CandidatusAmoebophilus	0.00	18.13
Sphingopyxis	0.26	17.92
Pseudomonas	5.15	10.84
Nevskia	20.02	8.21
Ochrobactrum	25.34	5.49
Halomonas	10.74	4.42
Vibrio	1.63	3.13
Kangiella	2.23	2.23
Hydrogenophaga	3.35	1.77
Shewanella	4.47	1.04
Serratia	4.72	0.53
Pannonibacter	0.26	0.30
Dietzia	1.12	0.30
Psychrobacter	0.69	0.15
Bacillus	0.09	0.06
Methylibium	0.09	0.06
Phaeospirillum	0.00	0.04
Methyloversatilis	0.09	0.04
Propionibacterium	0.00	0.04
Rhodanobacter	0.00	0.02
Azorhizobium	0.00	0.00
Afipia.1	0.00	0.00
Rhodococcus	0.09	0.00
Hydrocarboniphaga	0.09	0.00
